# SeqAcademy: an educational pipeline for RNA-Seq and ChIP-Seq analysis

**DOI:** 10.12688/f1000research.14880.4

**Published:** 2020-09-22

**Authors:** Syed Hussain Ather, Olaitan Igbagbo Awe, Thomas J. Butler, Tamiru Denka, Stephen Andrew Semick, Wanhu Tang, Ben Busby

**Affiliations:** 1 National Institute of Diabetes and Digestive and Kidney Diseases, National Institutes of Health, Bethesda, MD, 20892, USA; 2 National Center for Biotechnology Information, U.S. National Library of Medicine, National Institutes of Health, Bethesda, MD, 20894, USA; 3 National Institute on Aging , National Institutes of Health, Baltimore , MD, 21224, USA; 4 National Center for Biotechnology Information, National Institutes of Health, Bethesda, MD, 20894, USA; 5 Lieber Institute for Brain Development , Baltimore , MD, 21205, USA; 6 National Institute of Allergy and Infectious Diseases, National Institutes of Health, Bethesda, MD, 20892, USA

**Keywords:** RNA-Seq, ChIP-Seq, alignment, differential gene expression, peak-calling, education, tutorial, pipeline

## Abstract

Quantification of gene expression and characterization of gene transcript structures are central problems in molecular biology. RNA sequencing (RNA-Seq) and chromatin immunoprecipitation sequencing (ChIP-Seq) are important methods, but can be cumbersome and difficult for beginners to learn. To teach interested students and scientists how to analyze RNA-Seq and ChIP-Seq data, we present a start-to-finish tutorial for analyzing RNA-Seq and ChIP-Seq data: SeqAcademy ( *source code:* https://github.com/NCBI-Hackathons/seqacademy, *webpage:* http://www.seqacademy.org/). This user-friendly pipeline, fully written in markdown language, emphasizes the use of publicly available RNA-Seq and ChIP-Seq data and strings together popular tools that bridge that gap between raw sequencing reads and biological insight. We demonstrate practical and conceptual considerations for various RNA-Seq and ChIP-Seq analysis steps with a biological use case - a previously published yeast experiment. This work complements existing sophisticated RNA-Seq and ChIP-Seq pipelines designed for advanced users by gently introducing the critical components of RNA-Seq and ChIP-Seq analysis to the novice bioinformatician. In conclusion, this well-documented pipeline will introduce state-of-the-art RNA-Seq and ChIP-Seq analysis tools to beginning bioinformaticians and help facilitate the analysis of the burgeoning amounts of public RNA-Seq and ChIP-Seq data.

## Introduction

RNA sequencing (RNA-Seq) is a rapidly expanding technique used to answer broad questions in the life sciences, ranging from mitochondrial function ( Mercer *et al*., 2011) to the pathogenesis of breast cancer ( Li *et al*., 2017). Chromatin immunoprecipitation sequencing (ChIP-Seq) is a genome-wide technique for profiling histone modifications, DNA-protein interactions, and transcription factor binding sites ( Barski *et al*., 2007). Using this technique to analyze DNA-protein interactions involves very large data sets for computational analysis. The computational steps can identify the locations of features such as DNA-binding enzymes, modified histones, chaperones, nucleosomes, and transcription factors (TFs) ( Bailey *et al*., 2013).

The expanding importance of RNA-seq and ChIP-seq data is reflected by its explosive growth in terabytes in the primary public repository for storing this data - the Sequence Read Archive (SRA) ( Wheeler *et al*., 2008). This incredible increase in the amount of public data has not been met with an equal increase in the number of scientists who can skillfully and thoughtfully analyze this important resource. Given the fundamental role that RNA-seq and ChIP-seq data, among other next-generation sequencing data types, are likely to play in the coming decades, there is a critical need to teach RNA-seq and ChIP-seq analysis to life scientists with diverse interests and backgrounds.

The goal of analyzing RNA-seq data is often to identify and characterize quantitative differences in gene expression between biological samples from two or more groups. For ChIP-Seq, the goal is to characterize DNA-protein interactions. Biological samples may originate from several different study designs including: different tissue types from the same individual (e.g. cancerous tissue vs. non-cancerous tissue), the same strain of cells under different environmental conditions, or the same tissue under a time-course experiment.

There are major barriers to the novice bioinformatician who is interested in learning how to analyze RNA-Seq and ChIP-Seq data. RNA-Seq and ChIP-Seq data are costly to generate (>$1,000/sample) and cumbersome to store; with data from a single sample often occupying several gigabytes of storage space. However, recent advances, including a greater impetus to deposit sequencing data in SRA ( “Principles and Guidelines for Reporting Preclinical Research,” 2015) and the innovative alignment of streamed sequencing data ( Kim *et al*., 2015), offer new opportunities to overcome these long-standing problems. The second barrier to entry is inherent to RNA-Seq and ChIP-Seq data. These datasets are large and complex: there are over 20,000 known genes in the human genome ( Naidoo *et al*., 2011) and the transcriptional diversity of the human genome is not yet fully characterized ( Yamashita *et al*., 2011).

Furthermore, RNA-Seq data is susceptible to “batch effects” and other confounders that can occlude real biological effects or, worse, mislead the un-skeptical researcher. Thus, appropriate analysis of these data requires advanced algorithms and sophisticated statistical methods, coupled with traditional scientific skepticism, to uncover biological insight buried in the data.

These difficulties dissuade many from attempting RNA-Seq and ChIP-Seq analysis, particularly those lacking previous data analysis experience, but the genomics community needs more scientists who can adeptly analyze RNA-Seq and ChIP-Seq data. Moreover, shared understanding of RNA-Seq and ChIP-Seq analysis will produce higher quality discourse between the biologists who are responsible for conducting RNA-Seq and ChIP-Seq experiments and the bioinformaticians who are experts at analyzing the resulting data produced from these experiments. Several well-developed pipelines currently exist for processing RNA-Seq and ChIP-seq data from start to finish ( Djebali *et al*., 2017; Park *et al*., 2017; Torres-García *et al*., 2014; Yalamanchili *et al*., 2017); however, these pipelines are generally designed for advanced bioinformaticians who often have existing practical experience in analyzing high-throughput data. A pipeline designed to teach those with little experience how to analyze high-throughput sequencing data is therefore needed. Thus, we developed a proof-of-concept, well-documented “tutorial pipeline” over the course of a three-day NCBI-sponsored hackathon intended to teach RNA-seq and ChIP-seq analysis to beginners. This tutorial pipeline, “SeqAcademy,” incorporates state-of-the-art RNA-Seq and ChIP-seq analysis tools into a simple, easy to use workflow tutorial and we demonstrate its use with publicly available data.

## Methods

### Implementation

SeqAcademy uses self-contained tutorials, which runs Python, R, and Bash scripts among others, all from the document itself. It requires about 16 GB of memory storage. The tutorial files facilitate open science and reproducible code by mixing code chunks with notes and markup. This format, known as “literate programming,” is particularly amenable to teaching bioinformatics because it allows learners to follow along in the document while running each code step directly within the notebook.

### Operation

The tutorial begins with an explanation of how to install necessary dependencies and select interesting data from the BioProjects browser. Alignment while streaming the data is done with HISAT2 version 0.1.6 and subsequent quality control with MultiQC version 1.5. The tutorial then splits into two separate protocols: one for RNA-seq, the other for ChIP-seq analysis.

**Figure 1.**
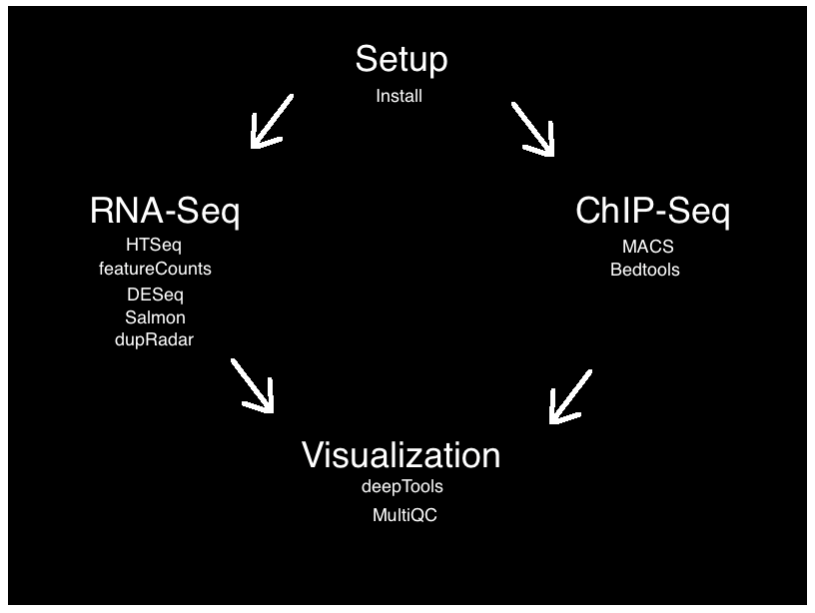
Flowchart of the SeqAcademy tutorial.

The workflow involved setup, alignment, quality control, analysis, and visualization steps for publicly available RNA-Seq and ChIP-seq data sets. There are many appropriate tools available for each step of RNA-seq and ChIP-seq analysis. Our goal is to present an easy to use and understandable pipeline rather than an exhaustive list of analysis tools. For each step below, we will explain the role of the bioinformatic tool, as well as our rationale for including it in this tutorial pipeline ( Figure 1). Here, we present an overview of the steps; further details for each subsection can be found on the project’s Github page.

### Setup

The setup step uses the Bioconda channel ( Grüning *et al*., 2017) for the conda package manager to install all of the programmatic dependencies for the entire pipeline. The data sets were selected by searching NCBI BioProject web browser ( Barrett *et al*., 2011). For our use case, we searched for publically available RNA-seq and ChIP-seq datasets that were relatively small and thus could be easily downloaded and processed, and would be relatively straightforward to interpret biologically. We therefore selected RNA-Seq and ChIP-Seq data from yeast ( *Saccharomyces cerevisiae)* samples ( Mulla *et al*., 2017; Rawal *et al*., 2018).

The RNA-seq data demonstrates the differences in genetic expression between aneuploid and euploid yeast ( Mulla *et al*., 2017). The ChIP-seq data demonstrates the effects of 3-Amino-1,2,4-triazole (3-AT) on chromatin accessibility ( Rawal *et al*., 2018). We downloaded the reference sequence for *Saccharomyces cerevisiae* from Ensembl version 84 (RNA-seq SRA study number: SRP106028 ChIP-seq SRA study number: SRP132584). We note that the *SraRunTables* file can be adjusted to specific user data, different from the RNA-seq or ChIP-seq data sets used in this project. Thus, this lightweight, portable educational pipeline can be adapted to meet the usage needs and interests of a broad base of bioinformatics beginners and teachers.

### Alignment

The purpose of alignment, map raw sequence reads to a reference genome, thereby allowing quantification of a genomic property (e.g. gene transcription in the case of RNA-seq). HISAT2 is a software program used for the alignment of raw sequence data, consisting of FASTQ files ( Kim *et al*., 2015. We chose to use HISAT2 because it allows users to stream raw sequence data rather than downloading it to the local machine, reducing disk space and time requirements for users of the SeqAcademy educational tool - an exemplary use of “edge-computing” in bioinformatics. One disadvantage of this approach is that it requires a stable internet connection, as the aligned raw sequence files are downloaded as SAM (sequence alignment mapping) files along with the log files. Nevertheless, by choosing to use HISAT2 for alignment, we reduced required disk space and broadened the potential user base of this pipeline.

### Quality control

Quality control is a critical step given that sequencing data is often of heterogeneous quality, and is a way of i) identifying outliers ii) assessing whether sequencing data is a valid measure of a genomic property To generate a quality control report about the success of the alignment, we used MultiQC ( Ewels *et al*., 2016). MultiQC reports the number of reads mapped to one unique location, reads mapped to multiple unique locations, and reads not mapped to any location in the reference genome. MultiQC can provide reports for both RNA-Seq and ChIP-seq data. Reads mapped to one unique location have a higher confidence level of being correctly mapped, as reads mapped to multiple unique locations cannot be localized to the reference with a high degree of probability. While MultiQC is not strictly necessary for this pipeline--the plots and statistics it produces are based off of the HISAT2 alignment summary files - we chose to include it to introduce users to a useful tool that is built for quality control.

### RNA-Seq

RNA-sequencing is a high throughput method for studying gene transcription. After alignment and quality control, users convert the SAM files to BAM files with the samtools package version 1.8 ( Li *et al*., 2009). Then, gene expression is quantified with HTSeq version 0.9.1 ( Anders *et al*., 2015). Quantification of gene expression is important for understanding transcript abundance and for making statistical comparisons of gene expression between groups.

Afterwards, we demonstrate how to extract biological significance from these various analyses, by showing students how to visualize gene expression patterns and undertake exploratory data analysis with principal component analysis. Principal component analysis (PCA) is an unsupervised clustering method best suited for studies including multiple samples. If only one RNA-seq sample is present, PCA is not an appropriate analysis as no dimensional reduction can be performed. For multiple samples with a single condition, PCA is a valuable tool for identifying and quantifying potential batch effects. When batch effects are successfully isolated by PCA, the corresponding batch PCs may be valuable as adjustment variables (i.e. covariates) in downstream analysis. For example, including batch PCs as covariates in differential gene expression analysis can help reduce confounding by batch. For multiple samples with multiple conditions, PCA can potentially distinguish groups and determine how much transcriptome-wide variance the condition explains. Notably, PCA offers a global picture of transcription and cannot determine which specific genes are different between conditions--individual genes are best identified via differential gene expression analysis (see DESeq2 below). Likewise, PCA may again be useful in this scenario for quantifying batch effects. Lastly, PCA of multiple samples can be used for as an additional quality control step with visual identification of outliers.

Finally, we show how to undertake differential expression analysis using DESeq2 version 1.21.0 ( Love *et al*., 2014) and how to visualize these differences with volcano plots and experiment-specific visualizations in the R package ggplot2 version 2.2.1 ( Wickham, 2009). Thus, students can learn how to quantify gene expression, answer biologically relevant questions through differential gene expression analysis, and visualize gene expression patterns.

### ChIP-Seq

After alignment, we perform peak-calling to determine protein-binding locations in the ChIP-seq data. The peak-calling step of ChIP-Seq involves finding differentially binding sites between the two ChIP-Seq signals (input and immunoprecipitate). Numerous peak callers exist to distinguish biologically relevant signal peaks from technical noise for the Chip-Seq experiments. Here, we used the peak-calling algorithm MACS (Model-based Analysis for ChIP-Seq) version 1.4.2 ( Zhang *et al*., 2008). MACS is a commonly used peak-caller and has been shown to have more accurate results than competing peak-callers ( Hocking *et al*., 2017). After calling peaks, the results are sorted and analyzed for intersections using bedtools version 2.27.0, a set of tools for analyzing genomic data ( Quinlan & Hall, 2010) with the genes annotated. Bedtools provides a set of tools for common genomics analysis techniques. It’s straightforward and popular within the field of bioinformatics. Lastly, bedtools output is visualized with Integrative Genomics Viewer (IGV) version 2.4, a genomic data set viewer that allows for visualization of genomic features ( Robinson *et al*., 2011).

## Use cases

### Target audience

This educational pipeline is designed for students without previous programming experience who are looking for an introduction to the acquisition, processing, analysis, and visualization of either RNA-seq or ChIP-seq data. Students of next-generation sequencing analysis may range the academic spectrum, from undergraduates to professors, all of whom share an interest in learning to analyze sequencing data. SeqAcademy also offers a useful introduction to the core steps of RNA/ChIP-seq analysis for use by bioinformatics educators who are teaching a class or mentoring students. Motivated individual learners, for instance a graduate student who is attempting RNA-seq analysis, may also benefit by working through SeqAcademy. The tutorial is completely self-contained, so users do not need to manage additional input files or tools beyond what is provided directly in the notebook document—every line of code to be run has already been written and tested. Thus, this flexible tutorial may be a suitable introduction to RNA-seq and ChIP-seq analysis for workshops, graduate school classes, or motivated individual learners. We also hope that fellow bioinformatics educators will build off of SeqAcademy to teach intermediate and advanced bioinformatics concepts and skills. The pipeline is simple and modular, so it can easily be adapted to analyze different datasets and customized to meet different user needs.

### Learning objectives

The learning objectives of SeqAcademy are two-fold. The first and most immediate or practical objective is for a student to learn how to conduct the core steps of an RNA/ChIP-seq analysis, beginning with a search for publicly available sequencing data and ending with biologically meaningful results. The second objective is to foster a greater understanding of the concepts behind each step. This includes biological reasons behind why certain experiments such as ChIP-Seq and RNA-Seq are run, and the logic behind alignment, differential gene expression, and peak-calling. The tutorial pipeline is purposefully simple, as this will introduce an important component of next generation sequencing more gently, and will encourage students to build off of it to create more advanced pipelines that will meet the unique goals of the student.

Table 1 and Table 2 illustrate the sample input yeast data for RNA-Seq and ChIP-Seq, respectively. The RNA-Seq data examines aneuploidy while the ChIP-Seq data shows induction by 3-Amino-1,2,4-triazole (3-AT). Results of the principal component analysis, an unsupervised data reduction technique, of the RNA-Seq data are shown in Figure 2a. The slight clustering of the data into two different groups, euploid and aneuploid can be observed. A volcano plot is used to visualize significant differentially expressed genes between two groups, in this case euploid and aneuploid ( Figure 2b). Figure 2c displays the enrichment of chromosome X for differentially expressed genes, consistent with the aneuploid sample having an extra X chromosome. Figure 3 shows an IGV screenshot of how peaks of protein-enrichment are distributed across the yeast genome. The corresponding genes can be examined to determine proteins involved in 3-AT induction.

**Table 1.** Example RNA-Seq input. This data presents the RNA-Seq data used in this tutorial. This tutorial observes RNA-Seq data of aneuploidy in yeast.

BioSample	Experiment	MBases	MBytes	Run	SRA_Study
SSAMN06859 211	SRX2775581	1632	575	SRR5494627	SRP106028
SAMN06859 210	SRX2775582	940	331	SRR5494628	SRP106028
SAMN06859 209	SRX2775583	1195	421	SRR5494629	SRP106028
SAMN06859 208	SRX2775584	815	288	SRR5494630	SRP106028
SAMN06859 207	SRX2775585	946	333	SRR5494631	SRP106028
SAMN06859 206	SRX2775586	1152	407	SRR5494632	SRP106028

**Table 2.** Example ChIP-Seq input. This data presents the ChIP-Seq data used in this tutorial. This tutorial observes ChIP-Seq data of induction by 3-AT in yeast.

BioSample	Experiment	MBases	MBytes	Run	SRA_Study
SAMN08513506	SRX3677830	8816	3690	SRR6703656	SRP132584
SAMN08513513	SRX3677835	9614	4022	SRR6703661	SRP132584
SAMN08513512	SRX3677836	6049	2749	SRR6703662	SRP132584
SAMN08513511	SRX3677837	6918	3140	SRR6703663	SRP132584

**Figure 2a.**
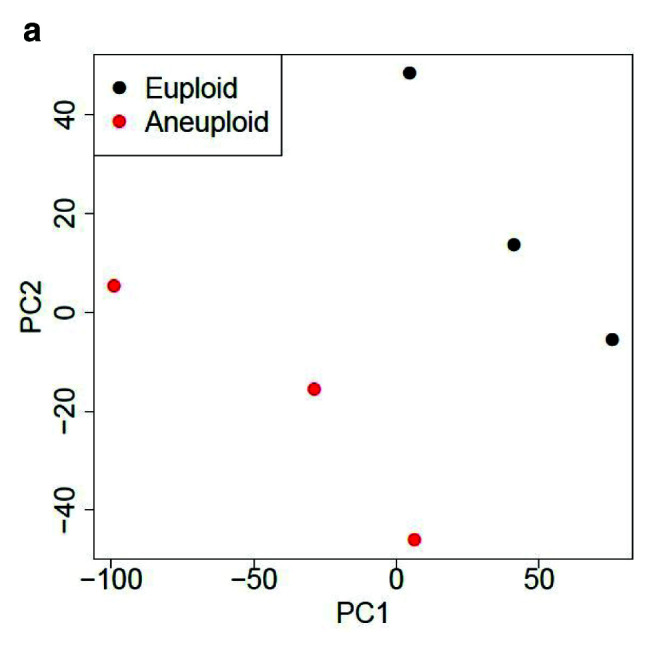
Principal component analysis (PCA) of yeast. PCA suggests gene expression for euploid yeast samples (haploid) clusters distinctly from that of the aneuploid yeast samples (diploid chromosome X). The first two Principal Components account for ~70% of the variance in expressed genes). Data provided by Mulla *et al.*, 2017.

**Figure 2b.**
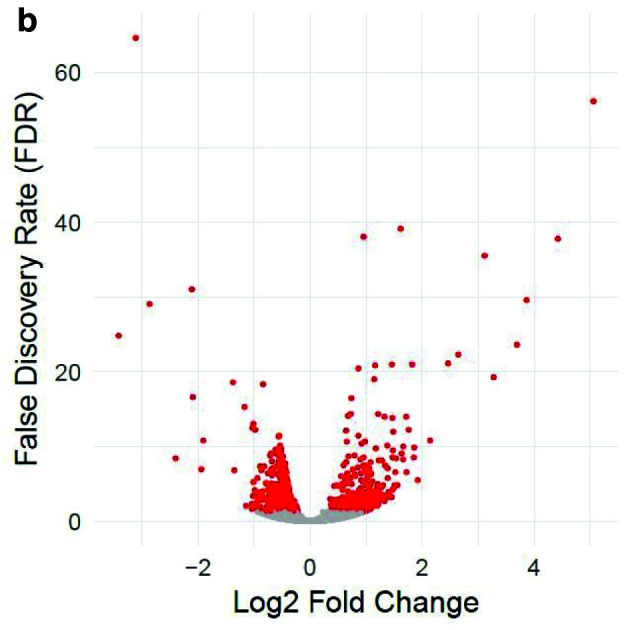
Volcano plot of differentially expressed genes between euploid yeast colonies versus aneuploid yeast colonies. The x-axis represents the difference in gene expression between the conditions. False discovery rate (FDR), a method for controlling for multiple testing, is along the y-axis. Each point represents a tested gene (N=3,926). Red points are those reaching genome-wide significance (at FDR<0.05, N=663), whereas grey points are genes not reaching statistical significance (FDR>0.05, N=3,263). Data provided by Mulla *et al.*, 2017.

**Figure 2c.**
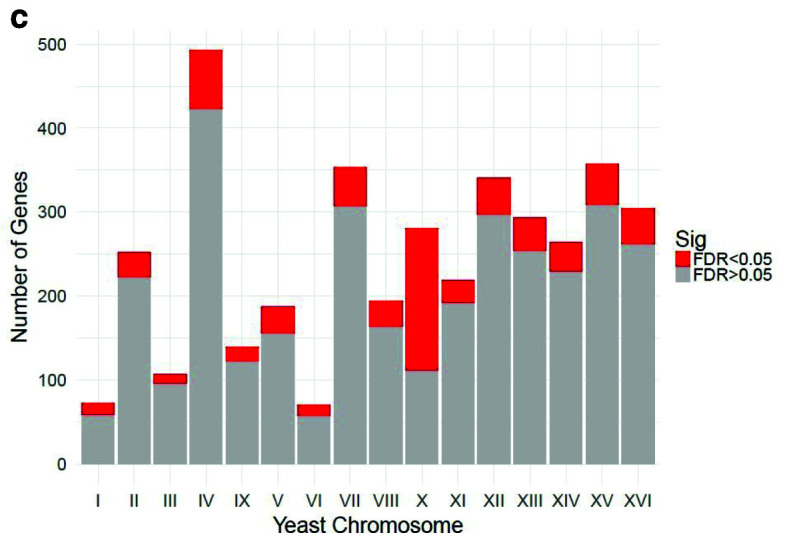
Relative enrichment of chromosome X for differentially expressed genes. The relative enrichment of chrX for differentially expressed genes suggests the downstream results of this processing pipeline are consistent with biological expectations. The RNA-seq experiment was performed on yeast colonies with an extra chromosome X. Data provided by Mulla *et al.,* 2017.

**Figure 3.**
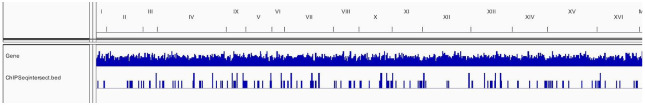
Distribution of intersected peaks across the yeast genome. This IGV screenshot shows in the bottom row the intersected peaks between the two treatment conditions of the yeast samples. The matching genes with each intersected peak can be analyzed. Data provided by Rawal *et al.*, 2018.

## Conclusion and next steps

### Limitations and future directions

There are several limitations to take into account with this tutorial and future directions for further work. In this tutorial, we focused on using RNA-seq on “bulk” or homogenate tissue samples, as opposed to single-cell RNA-seq, which has distinct analytical considerations. Our pipeline is currently limited to only two of the various next generation sequencing analyses, and we would like to broaden the scope to also include DNA sequencing and other epigenetic sequencing protocols, such as whole-genome bisulfite sequencing. Our platform can also be developed further to incorporate more advanced features, such user interfaces for performing bioinformatics analyses from the web browser, login systems for users to keep track of their own progress, and forums and messaging systems for community feedback. We would also like to translate the pipeline into other languages to broaden its scope. In subsequent improvements, we plan to make the pipeline easily individualized for a user’s own data sources by adjusting SraRunTables. Future hackathons may offer a useful setting to further improve this developing resource. Despite these limitations, SeqAcademy provides a solid starting foundation for beginners to learn the fundamentals.

### Summary

We have presented a novel, standalone educational tool for two types of next generation sequencing data: RNA-Seq and ChIP-Seq data. This project offers a simple guidebook to an introductory analysis pipeline used in RNA-Seq and ChIP-Seq data. We introduced a cutting-edge bioinformatics tools frequently used for the acquisition, alignment, processing, analysis, and visualization of large-scale sequencing data and referenced further resources for continued learning. SeqAcademy meets the need for an educational analysis pipeline which can be used to teach undergraduate and graduate students with limited bioinformatics experience how to analyze publically available sequencing data.

## Data availability

Use case data is available for the NCBI Sequence Read Archive Run Selector under accession numbers – SRP132584 and SRP106028

## Software availability

Archived source code as at time of publication: https://doi.org/10.5281/zenodo.2662541 ( Ather *et al.*, 2018)

The code for this project is deposited under an MIT License on GitHub: https://github.com/NCBI-Hackathons/seqacademy
