# Supplementary material for: SeqAcademy: an educational pipeline for RNA-Seq and ChIP-Seq analysis
Source: F1000Res. 2020 Sep 22;7:ISCB Comm J-628. Originally published 2018 May 22. [Version 4] doi: 10.12688/f1000research.14880.4 (PMC7525341; doi:10.12688/f1000research.14880.4)
Supplement: Peer review history

# Peer review: Reviewer response for version 4

**DOI:** 10.5256/f1000research.29651.r71788

**Published:** 2020-09-29

**Authors:** Xi Chen

**Affiliations:** 1 Center for Computational Biology, Flatiron Institute, Simons Foundation, New York, NY, USA

Overall I am satisfied with the updated text. The only thing they should revise is DNA-protein interaction. It is a directional binding event from protein to DNA, which can be captured using ChIP-seq. Therefore, it must be protein-DNA interaction. People sometimes call transcription factors as DNA-binding protein, however, DNA-protein interaction is a wrong concept.

Are the conclusions about the tool and its performance adequately supported by the findings presented in the article?

Yes

Is the rationale for developing the new software tool clearly explained?

Partly

Is the description of the software tool technically sound?

Partly

Are sufficient details of the code, methods and analysis (if applicable) provided to allow replication of the software development and its use by others?

Yes

Is sufficient information provided to allow interpretation of the expected output datasets and any results generated using the tool?

Partly

Reviewer Expertise:

NGS data analysis, computational tool development, bulk or single cell data integration, epigenetics, genetics

I confirm that I have read this submission and believe that I have an appropriate level of expertise to confirm that it is of an acceptable scientific standard.

# Peer review: Reviewer response for version 3

**DOI:** 10.5256/f1000research.20598.r49787

**Published:** 2019-06-13

**Authors:** Xi Chen

**Affiliations:** 1 Center for Computational Biology, Flatiron Institute, Simons Foundation, New York, NY, USA

I appreciate the efforts that the authors have made to make the RNA/ChIP-seq data processing easy by developing this pipeline. I can foresee the educational value of this paper. However, after reading this paper, I have several comments, especially about explaining ‘why’ each processing step is needed to users.

 It is not clear to me what’s the meaning of protein interactions. Are you talking about protein-DNA interactions or protein-protein interactions? ChIP-seq is mainly used to identify protein-DNA interactions. If multiple ChIP-seq profiles are jointly analyzed, co-binding relationship between multiple proteins can be studied. However, this co-binding relationship is different from protein-protein interaction. The author should say "protein-DNA interactions” instead of "protein interactions" as the latter usually refer to protein-protein interactions.

 After reading the abstract and introduction, it is not clear to me why RNA-seq and ChIP-seq data are jointly discussed in this paper. Most of the reference papers are just talking about one data type. Please clarify why you choose these two. I know people can integrate both data types to identify regulatory networks (doi: 10.1093/bioinformatics/btx827) ^1^. Is this your motivation? But this is not mentioned in this paper. As this manuscript is prepared to guide beginners lacking enough experience in this filed, it is necessary to tell them why these two data types are jointly discussed here. Although integrating these two data types is not the main focus of this paper, users can preprocess the raw data using this pipeline and do further downstream analysis by using other tools. Definitely, current description of each data type is necessary to let readers know each data type provides unique information.

 Quality Control should be placed right after alignment as it is a general approach to check read quality and alignment rate. It is very misleading to readers that they need to get peaks or gene expression and then do QC check on BAM files.

 The following part of RNA-seq data analysis should be improved “Afterwards, we demonstrate how to extract biological significance from these various analyses, by showing students how to visualize gene expression patterns and undertake exploratory data analysis with principal component analysis (PCA). Finally, we show how to undertake differential expression analysis using DESeq2 version 1.21.0 (Love et al., 2014) and how to visualize these differences with volcano plots and experiment-specific visualizations in the R package ggplot2 version 2.2.1 (Wickham, 2009).” Users may have their own data and it can be: (1) one sample, (2) multiple sample under one condition, (3) multiple sample under two conditions. It would be better to discuss processing steps in different scenarios. If only one RNA-seq sample is given, there is no need to do PCA or DESeq2. For (2), PCA can tell if there is batch effect. For (3), PCA can tell how different (at high level) those samples are between two conditions and further DESeq2 can tell which genes are different. In this paragraph, the authors say a lot of ‘how’, but to beginners, they need to know ‘why’ each step is needed.

 The same issue to ChIP-seq data. Why do users need the intersection step using bedtools? If they get multiple replicates, the intersection step will return reliable peak loci. If data of multiple factors is obtained, they can use the interaction step to extract co-binding events. And gene annotation is usually needed after getting those peaks, which is missing in this pipeline.

Are the conclusions about the tool and its performance adequately supported by the findings presented in the article?

Yes

Is the rationale for developing the new software tool clearly explained?

Partly

Is the description of the software tool technically sound?

Partly

Are sufficient details of the code, methods and analysis (if applicable) provided to allow replication of the software development and its use by others?

Yes

Is sufficient information provided to allow interpretation of the expected output datasets and any results generated using the tool?

Partly

Reviewer Expertise:

NGS data analysis, computational tool development, bulk or single cell data integration, epigenetics, genetics

I confirm that I have read this submission and believe that I have an appropriate level of expertise to confirm that it is of an acceptable scientific standard, however I have significant reservations, as outlined above.

## References

[1] : CRNET: an efficient sampling approach to infer functional regulatory networks by integrating large-scale ChIP-seq and time-course RNA-seq data. *Bioinformatics*.2018;34(10) : 10.1093/bioinformatics/btx827 1733-1740 10.1093/bioinformatics/btx827 PMC594687629280996

# Peer review: Reviewer response for version 2

**DOI:** 10.5256/f1000research.18855.r41334

**Published:** 2019-03-11

**Authors:** Philip Ewels

**Affiliations:** 1 Stockholm University, Department of Biochemistry and Biophysics, Stockholm, Sweden

The authors have made significant improvements to the tool in this revision, addressing a number of points raised in my initial review. However, sadly I was still unable to get the tutorial code to work myself. A number of the steps generated syntax and execution errors making progress impossible. The notebook has some statuses from the authors' previous execution steps with similar error messages, so I don't think that this is just my system (downloading the genome & building the index onwards).

 There seems to be some active development work ongoing in the repository - both a `devel` workbook (better to use git branches instead of separate files), but also some duplicate code blocks in the main notebook. There also seems to be a discrepancy between the introduction text on the main website and on the repository.

 In summary - I still think that the intention and design of the tool is excellent, and the manuscript text good. However, whilst the tutorial itself seems to be broken and not usable by others, I cannot approve this article. If these issues can be fixed so that it's possible for others to easily run through the tutorial, then I will be happy to approve.

Are the conclusions about the tool and its performance adequately supported by the findings presented in the article?

Partly

Is the rationale for developing the new software tool clearly explained?

Yes

Is the description of the software tool technically sound?

Yes

Are sufficient details of the code, methods and analysis (if applicable) provided to allow replication of the software development and its use by others?

Yes

Is sufficient information provided to allow interpretation of the expected output datasets and any results generated using the tool?

Yes

Reviewer Expertise:

NA

I confirm that I have read this submission and believe that I have an appropriate level of expertise to state that I do not consider it to be of an acceptable scientific standard, for reasons outlined above.

# Peer review: Reviewer response for version 1

**DOI:** 10.5256/f1000research.16196.r34336

**Published:** 2018-06-12

**Authors:** Norann A. Zaghloul

**Affiliations:** 1 Department of Medicine, Division of Endocrinology, Diabetes and Nutrition, Program in Personalized and Genomic Medicine, University of Maryland School of Medicine, Baltimore, MD, USA

This article presents a streamlined tool aimed toward novice bioinformaticians who wish to analyze RNA-Seq or ChIP-Seq data in one simple tool. The premise for this tool is excellent. Investigators who are new to these approaches often find the analysis of data daunting and are unsure of the tools to use for each step. SeqAcademy provides a single tool for all the steps in an easy to download package. Given that the target for this tool is investigators who do not have extensive expertise in the tools and analyses available, the article would benefit from more detailed descriptions of some individual components. Specifically, the following:

 1. Alignment: The rationale for use of HISAT2 is clearly described. The time needed for this step should be discussed and, if possible, it would be helpful to provide an alternate local tool that could be used if users are unable to maintain consistent internet connectivity over the time needed for alignment completion.

 2. RNA-Seq: A critical piece of RNA-Seq analyses are the statistical tools used to define differential expression. Some brief discussion of important cut-offs would be helpful.

 3. Most tools incorporated into the package are described in 1-2 sentences. This should be consistent throughout, including for HTSeq, DESeq2.

 The tool is really quite useful and I believe will be very valuable to novice investigators and students learning the pipeline of RNA-Seq or ChIP-Seq. Given that, it would be very helpful to provide more, not less, explanation of some of the basic tools such that the educational goal of this package will be served.

Are the conclusions about the tool and its performance adequately supported by the findings presented in the article?

Yes

Is the rationale for developing the new software tool clearly explained?

Yes

Is the description of the software tool technically sound?

Yes

Are sufficient details of the code, methods and analysis (if applicable) provided to allow replication of the software development and its use by others?

Yes

Is sufficient information provided to allow interpretation of the expected output datasets and any results generated using the tool?

Yes

Reviewer Expertise:

NA

I confirm that I have read this submission and believe that I have an appropriate level of expertise to confirm that it is of an acceptable scientific standard.

# Peer review: Reviewer response for version 1

**DOI:** 10.5256/f1000research.16196.r34335

**Published:** 2018-06-05

**Authors:** Philip Ewels

**Affiliations:** 1 Stockholm University, Department of Biochemistry and Biophysics, Stockholm, Sweden

Bioinformatics training is of critical importance for the field of genomics, especially given the current shortage of experienced bioinformaticians and the utility of cross-training lab scientists in data analysis. In this manuscript, the authors describe a training tool developed to help teach people new to bioinformatics how to handle data from RNA and ChIP high throughput sequencing experiments.

 Such a tool is valuable to the genomics community - it is a resource for interested parties to use in their own time and could also be very useful for those designing bioinformatics courses.

**### Manuscript**

 The manuscript does a good job of describing the resource, as well as the reason it is helpful. In my opinion, it is longer than needed - the Introduction could be more concise and the *Use cases* section would feel more natural in the introduction. The figures could also easily be condensed into one and the table be a supplementary figure. Making the manuscript shorter would make it more accessible.

**### Tutorial**

 The authors take an excellent approach to the design of their tutorial, using a Jupyter notebook and software installations from bioconda. These are appropriate, user friendly and provide an excellent starting point for well documented and reproducible analyses. Some dependencies are required, but these are well described on the tool's homepage and commonly used in bioinformatics. I attempted to run the tutorial on my laptop, but I ran into a few issues that required some debugging.

**Broken steps**

 I came across a couple of problems in the notebook: I couldn't get the MultiQC commands to work - the mix of Python variables in the bash commands failed in my Jupyter notebook. I changed the command to use the following command:

 !multiqc test/{"* test/".join(RNASeqoutrun)}* --quiet --outdir test/multiqc_rnaseq --force

 Next, the Bedtools steps didn't work - the intersect command refers to a sample SRR6703663, which does not seem to be included anywhere in the tutorial before this point. I gave up at this point, so I'm not sure about the final steps. If the authors think that I did something wrong, I'd be happy to have another go.

 I recommend trying to get some colleagues to run this tutorial from scratch to get more feedback.

**External scripts**

 My main concern with the tutorial is that much of the downstream processing is done within R scripts outside of the Jupyter notebook. For me, this sort of misses the point - it would be preferable to have this code mixed in with the tutorial without any external scripts. It should be possible to have both Python (bash) and R in the same notebook, and this would make those steps part of the exercise rather than an abstract *"runDeseq.R"* script that requires the user to go and find and read through.

**Data subsampling**

 Although the authors have endeavoured to use a small dataset (Yeast, not Human), the data used is still large. The authors note in the tutorial that the alignment *"will most likely take several hours."*, which limits the utility of the tutorial in any teaching scenario.

 It should be quite easy to use the -u/--qupto  flag in HiSAT2 to limit the number of alignments performed. I imagine that most of the downstream analysis will still work with subsampled data, and will run much faster. I tried doing this with 1,000,000 reads and the alignment step completed in a couple of minutes.

**### Tutorial - Minor points**

**Software requirements**

 I had trouble getting the bioconda installation command to first install and then activate inside the the notebook. I would instead recommend creating the conda environment on the terminal first (including jupyter as a package to install), activating it and then running jupyter. This is what I ended up doing to get the tutorial to work.

**Streaming Input Data**

 The approach of streaming data from the SRA for the alignment step is a little non-standard. The reasoning is described in the paper as helping to reduce the disk space footprint required for the analysis. Whilst its cool that this step works, I would argue that it lessens the value of the tutorial, as most bioinformatics projects can not use such a method. Starting with regular static FastQ files would be more typical and useful for future analyses. This would also allow the additional step of FastQC for raw data analysis, which is pretty typical for such pipelines. The downside of this is that it invalidates my above subsampling idea! But SRA data could still be streamed to FastQ files with a similar subsampling approach.

**Two-in-one analysis**

 The analysis presented uses common alignment and quality control steps for both sets of data, before splitting into bespoke RNA and ChIP-seq analysis. I see the reasoning for this but I think this complicates issues a little for users. Personally, I would prefer to see two separate Jupyter notebooks and separate analyses for the two different data types to avoid confusing the two. This would simplify future expansion of the resource across additional data types, which may not be able to share such analysis steps.

**MultiQC reporting**

 The Quality Control step using MultiQC is placed after the HiSAT2 alignment. It would be better placed at the end of the analysis, as MultiQC also supports outputs from MACS, HTSeq and deepTools. A more complete report would be generated if it were run with all of these outputs.

**### Conclusions**

 I think that the aim of this paper is worthwhile and the approach is very nice. The tutorial itself feels a little unpolished and could do with a few trial runs on willing volunteers to iron out the issues I encountered. With a few tweaks and the changes above, I think that this tutorial could be the beginning of an excellent resource for budding bioinformaticians and educators.

Are the conclusions about the tool and its performance adequately supported by the findings presented in the article?

Partly

Is the rationale for developing the new software tool clearly explained?

Yes

Is the description of the software tool technically sound?

Yes

Are sufficient details of the code, methods and analysis (if applicable) provided to allow replication of the software development and its use by others?

Yes

Is sufficient information provided to allow interpretation of the expected output datasets and any results generated using the tool?

Yes

Reviewer Expertise:

NA

I confirm that I have read this submission and believe that I have an appropriate level of expertise to state that I do not consider it to be of an acceptable scientific standard, for reasons outlined above.
